# Placental Leucine Aminopeptidase as a Potential Specific Urine Biomarker for Invasive Ovarian Cancer

**DOI:** 10.3390/jcm11010222

**Published:** 2021-12-31

**Authors:** Tetsuya Matsukawa, Shigehiko Mizutani, Kunio Matsumoto, Yukio Kato, Masato Yoshihara, Hiroaki Kajiyama, Kiyosumi Shibata

**Affiliations:** 1 Department of Obstetrics and Gynecology, Fujita Health University Bantane Hospital, Aichi 454-8509, Japan; matsukawa.t1@med.nagoya-u.ac.jp; 2 Department of Obstetrics and Gynecology, Nagoya University Graduate School of Medicine, Aichi 466-8550, Japan; kajiyama@med.nagoya-u.ac.jp; 3 Daiyabilding Lady’s Clinic, Aichi 451-0045, Japan; daiya-lc@amber.plala.or.jp; 4 Division of Tumor Dynamics and Regulation, Cancer Research Institute, Kanazawa University, Kanazawa 920-1192, Japan; kmatsu@staff.kanazawa-u.ac.jp; 5 Department of Molecular Pharmacotherapeutics, Faculty of Pharmacy, Kanazawa University, Kanazawa 920-1192, Japan; ykato@p.kanazawa-u.ac.jp

**Keywords:** leucine aminopeptidase, ovarian cancer, urine biomarker, placenta

## Abstract

Background: A non-invasive and sensitive biomarker for the detection of ovarian cancer (OvCa) is lacking. We aim to investigate if urinary placental leucine aminopeptidase (P-LAP) can serve as a reliable biomarker for OvCa. Methods: P-LAP activity was measured using a LAP assay kit (Serotech Co., Ltd., Sapporo, Japan) in the urine of 22 patients with benign or borderline malignant ovarian tumors and 18 patients with OvCa. In this assay, L-methionine was added at 20 mM because P-LAP is functional, but other aminopeptidases are inhibited at this dose of L-methionine. Results: The mean urinary P-LAP activity was significantly higher in the OvCa group than in the benign or borderline malignant tumor group. When the cut-off value of P-LAP was determined as 11.00 U/L, its sensitivity and specificity for differentiating invasive cancer were 77.8% and 95.5%, respectively. Conclusion: Although the usefulness of this test should be confirmed in a larger cohort of cases and controls, our study is the first to highlight the importance of urinary P-LAP as a biomarker for OvCa.

## 1. Introduction

The identification of novel biomarkers has advanced ovarian cancer (OvCa) research and definitively contributed to the treatment for the disease. Currently, blood biomarkers are widely used for the diagnosis of ovarian cancer; however, these require an invasive process for sample collection. Therefore, the discovery of non-invasive and sensitive biomarkers used to detect OvCa is highly desirable.

Placental leucine aminopeptidase (P-LAP) (EC.3.4.11.3) is an endolysosomal enzyme synthesized as a membrane-spanning cysteine aminopeptidase [1,2,3]. P-LAP is expressed in various tissues, including the placenta. Notably, P-LAP is identical to insulin-regulated membrane aminopeptidase [4], which colocalizes with glucose transporter type 4 in the intracellular vesicles of fat and muscle cells. We found that P-LAP plays an important role in regulating feto-placental circulation to maintain physiological maternal blood pressure [5,6,7]. We also reported that P-LAP is expressed in the ovarian epithelium [8] and dysgerminoma of the ovary [9] and that blood P-LAP levels increase in patients with different types of gynecological malignancies [10]. P-LAP is present in endometrial adenocarcinoma and participates in the regulation of endometrial cancer growth [11]. Furthermore, P-LAP concentration assessed by immunohistochemical staining is associated with a poor prognosis in patients with endometrial adenocarcinoma [12]. On the other hand, P-LAP is also expressed in the kidney [13,14], and P-LAP activity increases in patients with high-grade and advanced-stage clear cell renal carcinoma [15,16].

The significant increases in P-LAP levels and activity among patients with gynecological and kidney malignancies prompted us to quantify P-LAP activity in the urine of patients with OvCa. We report that urine P-LAP activity significantly and sensitively increases in patients with OvCa compared with controls, which nominates urine P-LAP as a sensitive and non-invasive biomarker for detecting OvCa.

## 2. Patients and Methods

Patients who visited our institution and were diagnosed with primary ovarian tumor were included for this study. Written informed consent was received from all individuals whose urine and blood were provided. We included patients who visited our hospital from 2018 to 2020 with agreement for the study enrollment and excluded patients who had severe organ failure.

Measurement of P-LAP activity: P-LAP activity was measured using a LAP assay kit (Serotec Co., Ltd., Sapporo, Japan). In this assay, L-methionine was added at 20 mM because P-LAP is functional while other aminopeptidases are inhibited at this dose of L-methionine [17], allowing for the selective measurement of P-LAP activity even in the presence of other aminopeptidases in the urine sample. The determination of the cancer antigen 125 (CA125): Serum CA125 level was measured by an out-sourced institute. To compare changes in the urinary P-LAP activity and serum CA125/CA19-9 levels, urine and serum samples were collected from a patient (*n* = 1) with relapse before and after surgery.

Statistical analyses between two groups were performed using a Mann–Whitney U test for continuous variables. The difference in ROC curves between the groups was assessed by DeLong’s test. Significance was selected as two-sided with a *p* value < 0.05. All statistical analyses were conducted using IBM SPSS Statistics, Version 26.0 (IBM Corp., Armonk, NY, USA).

## 3. Results

**Figure 1:**
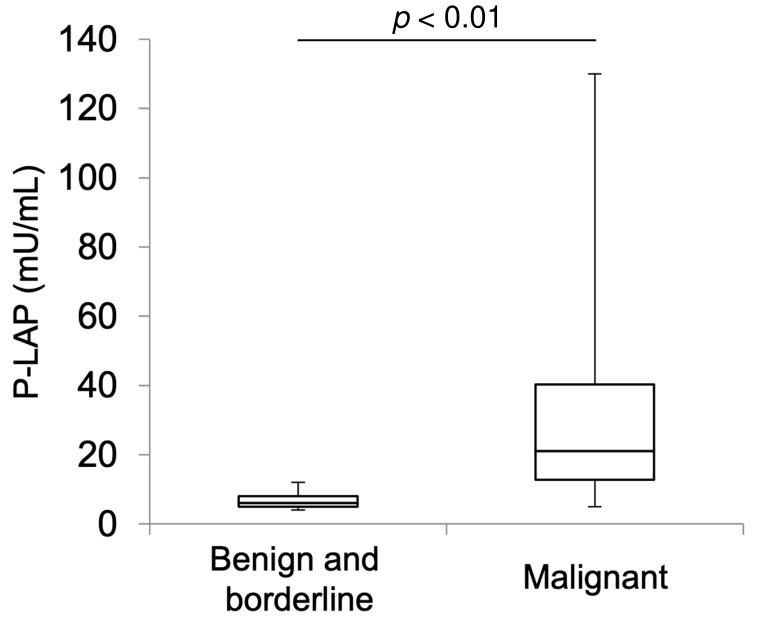
Box plot summary of P-LAP activity in the urine of 22 patients with benign or borderline malignant ovarian tumors and 18 patients with OvCa.

**Figure 2:**
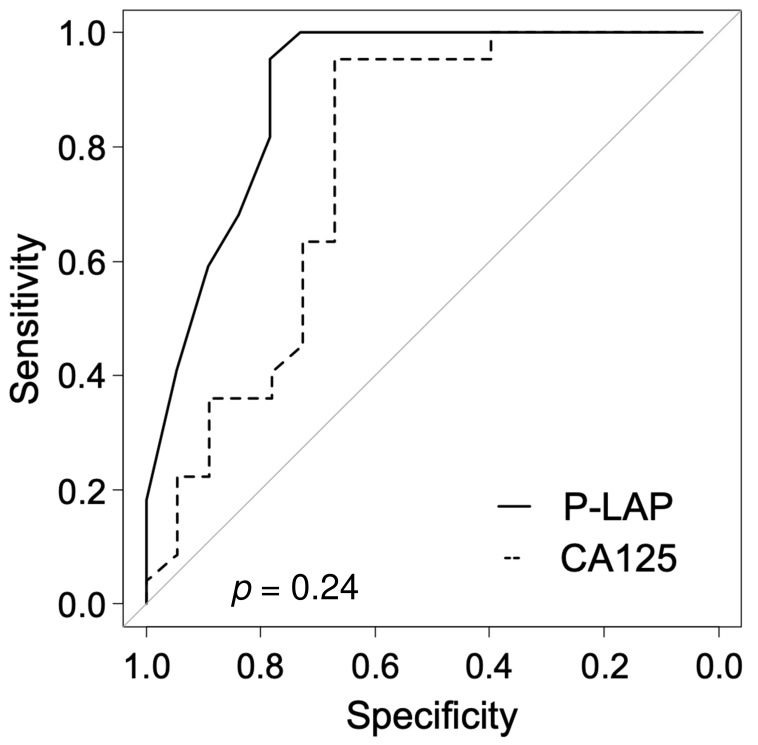
Receiver operating characteristics of urinary P-LAP and serum CA125.

We measured urinary P-LAP activity and serum levels of CA125 in 22 patients with benign or borderline malignant ovarian tumors and 18 patients with OvCa (Table 1). In patients with benign or borderline malignant tumors, chocolate cysts were observed in 11 patients (50%), and borderline malignant tumors were observed in three patients. Among the patients with OvCa, seven had serous carcinoma and five had clear cell carcinoma. In the benign/borderline malignant tumor group, the mean urinary P-LAP activity was 6.59 ± 2.32 U/L. In the OvCa group, urinary P-LAP activity was 30.61 ± 29.55 U/L; the value was significantly higher in the latter (*p* < 0.01; Mann–Whitney U test) (Figure 1). When the cut-off value of P-LAP was determined as 11.00 U/L, its sensitivity and specificity for identifying OvCa reached maxima of 77.8% and 95.5%, respectively. The area under the curve (AUC) of the sensitivity vs. specificity plot of P-LAP was larger than that of CA125, but the difference was not significant (*p* = 0.24; DeLong’s test) (Figure 2).

In one patient, we observed changes in their P-LAP activity and serum CA125/CA19-9 levels during treatment. The patient was 53-year-old woman. She had ovarian clear cell carcinoma with a maximum diameter of 12 cm and stage ⅡB ovarian cancer (FIGO 2014). Surgery including total hysterectomy, bilateral salpingo-oophorectomy, omentectomy, and lymph node biopsy, and two courses of paclitaxel and carboplatin therapy as postoperative adjuvant therapy were performed. Three months after the end of treatment, recurrence was confirmed by CT. Chemotherapy was not effective, and the patient died 9 months later.

**Figure 3:**
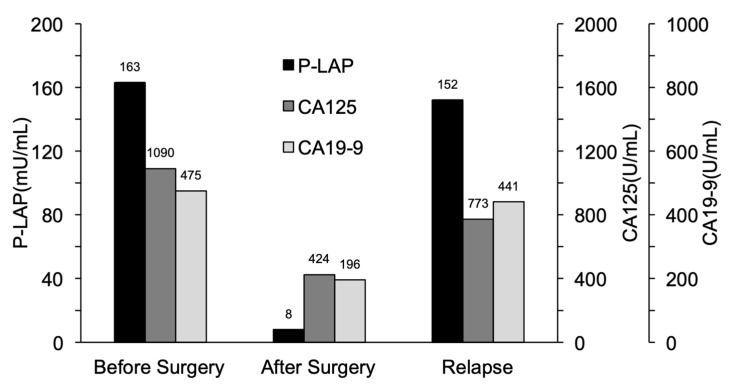
Temporal profiles of urinary P-LAP, serum CA125, and CA19-9 in a patient with post-surgical relapse of OvCa.

The urinary P-LAP activity and serum CA125/CA19-9 levels were determined during treatment (Figure 3). Both values were high before surgery but decreased after surgery, suggesting that these parameters were responsive to treatment. The rate of decrease in the urinary P-LAP activity was greater than that of the serum CA125 and CA19-9 levels. In addition, there were increases in the serum CA125 and CA19-9 levels, which were possibly related to tumor relapse after surgery. However, the rate of increase in the urinary P-LAP activity was greater than that of the serum CA125 and CA19-9 levels.

## 4. Discussion

Serum CA125 is often measured in cases of ovarian cysts to exclude their malignancy. However, because of its low specificity and increased levels in different physiological states, serum CA125 is not a good biomarker for OvCa [18]. Several benign conditions can cause elevations in CA 125, including pregnancy, uterine fibroids (benign tumors), normal menstruation, and pelvic inflammatory disease. CA 125 levels can also be elevated in endometriosis, a common disease in women of reproductive age and characterized by ovarian cysts. However, these cysts are usually benign and, only in some cases, can undergo malignant transformation. Therefore, the discovery of specific biomarkers is necessary to enable the early diagnosis of OvCa.

Cellular transformation has long been associated with increased secretion of lysosomal enzymes, a process that has been proposed to play a role in cancer growth and metastasis [19,20]. P-LAP is synthesized and localized as a lysosomal enzyme [1,3,21]. However, the mechanisms by which urinary P-LAP levels increase in patients with OvCa are largely unknown. There seem to be two possibilities: (1) P-LAP may be derived from malignant cancer cells, and (2) P-LAP may be derived from the kidney through communication by cancer cell-derived mediators. In the former case, during progression from benign to malignant characteristics, secretory characteristics of lysosomal P-LAP from tumor cells may be sensitively changed, which eventually results in an increase in urine P-LAP. On the other hand, P-LAP is abundantly expressed in various organs, including renal tubules and collecting tubules in the kidneys [13,14] and can be selectively released in response to a tumor at a distant site.

Exosomes are a subset of extracellular vehicles that originate from the endosomal system and are released in the extracellular milieu [22]. Considering recent advances in our understanding of the vital roles of exosomes in systemic communication between tumor cells and other organs, exosome-mediated communication between malignant ovarian tumor cells and host renal cells may potentially influence the secretory processes of P-LAP from renal cells [23,24,25,26,27].

Proteomic analysis of vesicles isolated from urine has identified numerous components of multivesicular bodies, which are a subset of endosomes that contain membrane-bound intraluminal vesicles as well as aminopeptidases such as aminopeptidase N and A [28]. Follow-up studies are needed to examine if proteins from such vesicles in patients’ urine can serve as robust biomarkers for other diseases.

One limitation of our study is that our sample size was small; therefore, patients with borderline ovarian tumors were included in the control group. However, P-LAP activity still differentiated between the control group, including non-invasive borderline cases, and patients with OvCa with high sensitivity and specificity. This highlights the superiority of this test for identifying the change from borderline to cancer. In addition, the size of the tumor was missing, which might affect the level of P-LAP. Furthermore, urine is a more favorable body fluid for biomarker detection because of its ease of collection, available volume, and protein profile that is less complex than that of plasma or serum.

To our knowledge, this study is the first to test and identify urine P-LAP activity as a biomarker for OvCa. The robustness and effectiveness of P-LAP activity as a biomarker should be assessed in larger validation cohorts in the future.

## 5. Conclusions

In conclusion, this study found that P-LAP activity may serve as a potential urine biomarker for detecting OvCa.

## Figures and Tables

**Table 1:** Urinary P-LAP levels and serum CA125 levels in ovarian tumor patients by histological types and stages.

		P-LAP (uU/mL)	CA125 (U/mL)
		Mean ± SD	Mean ± SD
Benign and borderline (*n* = 22)		6.59 ± 2.32	32.32 ± 24.98
Histology	Endometriotic Cyst (*n* = 11)	6.27 ± 1.68	30.64 ± 16.86
	Mature Teratoma (*n* = 6)	5.67 ± 2.73	37.33 ± 40.47
	Serous adenoma (*n* = 1)	12	56
	Mucinous adenoma (*n* = 1)	5	11
	Mucinous borderline (*n* = 2)	8.00 ± 0.00	37.00 ± 16.97
	Granulosa cell tumor (*n* = 1)	9	9
Stage	I (*n* = 3)	8.33 ± 0.58	27.67 ± 20.13
Malignant (*n* = 18)		30.61 ± 29.55	175.50 ± 256.32
Histology	Serous Carcinoma (*n* = 7)	27.14 ± 17.56	94.43 ± 83.37
	Clear Carcinoma (*n* = 5)	57.60 ± 41.84	416.20 ± 405.41
	Endometrioid Carcinoma (*n* = 3)	12.67 ± 6.81	52.00 ± 38.20
	Mucinous Carcinoma (*n* = 2)	7.00 ± 1.41	121.50 ± 47.38
	Carcinosarcoma (*n* = 1)	21	18
Stage	I (*n* = 6)	10.67 ± 5.20	81.17 ± 46.94
	II (*n* = 7)	45.29 ± 39.08	297.43 ± 385.53
	III (*n* = 5)	34.00 ± 19.96	118.00 ± 100.11

P-LAP-placental leucine aminopeptidase; CA-cancer antigen.

## Data Availability

Data are available from the authors upon reasonable request and with permission by Fujita Health University Bantane Hospital.
